# The Treatment of Complementary and Alternative Medicine on Female Infertility Caused by Endometrial Factors

**DOI:** 10.1155/2022/4624311

**Published:** 2022-09-07

**Authors:** Jing Lin, Haoyue Ma, Hang Li, Jing Han, Tingting Guo, Zhen Qin, Liyan Jia, Yuehui Zhang

**Affiliations:** ^1^Chinese Medicine Department, The Second Affiliated Hospital of Harbin Medical University, Harbin, China; ^2^Heilongjiang University of Chinese Medicine, Harbin, China; ^3^Department of Obstetrics and Gynecology, The First Affiliated Hospital, Heilongjiang University of Chinese Medicine, Harbin, China

## Abstract

Recently, with the development of the social economy, the incidence of infertility has increased year by year. With its complex etiology and diversified syndromes, infertility has become one of the most important diseases that plague the physical and mental health of women of childbearing age worldwide. Endometrial factors as an important part affecting female reproductive capacity, due to which induced repeated abortion and multiple uterine cavity operations occur, can destruct endometrium, failing to provide a normal implantation environment for zygote, thus resulting in infertility. Many patients failed to achieve expected results after receiving conventional treatments such as hormone therapy, assisted reproductive technology (ART), granulocyte colony-stimulating factor (G-CSF) therapy, and cell therapy, then turn to complementary and alternative medicine (CAM) therapies for help. Aiming at clarifying the effectiveness and mechanisms of CAM therapy in the treatment of infertility caused by endometrial factors, our paper systematically searched and studied present related literature on the PubMed, CNKI, and other databases, focusing on the aspects of clinical application and mechanism explorations and highlighting the therapeutic effects of Chinese herbal medicine (CHM), acupuncture, and moxibustion on such diseases. Moreover, this paper also introduces the CAM treatments of traditional Chinese medicine (TCM) retention enema, neuromuscular electrical stimulation (NMES), photobiomodulation therapy, dietary intervention, and other measures for infertility caused by endometrial factors, in order to provide a reference for subsequent basic research and clinical work.

## 1. Introduction

Infertility is defined as a multifactorial and complex reproductive system disorder in which a couple fails to establish a clinical pregnancy after 12 months of normal unprotected intercourse [1]. Biological, genetic, reproductive tract infection, lifestyle, and environmental factors are all associated with female reproductive capacity [2–4]. Infertility has affected 8%–12% of couples of childbearing age globally, with the female factors accounting for nearly as much as the male factors [3, 5]. According to the statistics, the incidence of infertility among women of childbearing age is about 14.3% in Western countries, about 25% in developing countries, and even as high as 30% in backward countries [6].

Infertility can be caused by different diseases, such as ovulation dysfunction, fallopian tube diseases, and uterine/peritoneal diseases, among which the uterine/peritoneal diseases account for about 10% of the common causes of infertility [7, 8]. Normal endometrial function is one of the necessary conditions for successful conception of women. However, the abnormalities of endometrium, such as thin endometrium, endometritis, and adenomyosis, can induce the occurrence of infertility, by altering the endometrial glycosylation patterns, adjusting the gene expression level of endometrium-related factors, or enhancing local endometrial estrogen effect, which directly reduce the endometrial receptivity that affects the implantation of zygote [9–12]. For patients with infertility caused by endometrial factors, drug or surgical treatment is often required. Nevertheless, the gonadotropin-releasing hormone agonists can cause changes in related hormone levels, leading to severe ovarian hyperstimulation syndrome (OHSS) and adversely affecting endometrial receptivity [13, 14]. OHSS is also the most serious complication of ART, which has a high failure rate and even life-threatening effect [15]. G-CSF promotes endometrial thickening and improvement of endometrial receptivity, affects embryo implantation, and fundamentally reduces pregnancy loss [16]. Additionally, cell therapy, such as platelet-rich plasma therapy and stem cell therapy, improves endometrial receptivity and clinical pregnancy rates by inducing the production of endometrial cytokines [17, 18]. G-CSF therapy and cell therapy, as emerging methods for the treatment of infertility in recent years, are costly. And their safety remains to be verified by larger-scale clinical trials. Therefore, many patients resort to CAM for treatment in order to obtain higher efficacy and safety.

Current studies have shown that CAM is widely used for infertile women in the world full of regional and cultural background differences [1]. For instance, CAM was used by approximately 63.5% of infertile patients in South Korea, 51% in Turkey, and 49.6% in Iran [19–21]. Long-term treatment with modern medicine has had a huge negative impact on the patients' psychological status and quality of life. As an important supplement to mainstream medicine, CAM is being increasingly chosen and recognized by growing infertile patients who hope to relieve mental stress and improve fertility outcomes through CAM treatment, for its few side effects and high security [22, 23]. Apart from that, as an important cause of infertility, the treatment of endometrial factors involves many CAM treatment methods, but it is still in the exploratory stage. This article focuses on reviewing the intervention measures and mechanisms of CAM in infertility caused by endometrial factors, in order to provide guidance and reference for further research and treatments.

## 2. Methods

Literatures related to the effectiveness and mechanism of CAM in the treatment of infertility caused by endometrial factors in the past five years have been systematically searched and studied in the PubMed, CNKI, and other databases. Data from studies on the CAM in the treatment of infertility caused by endometrial factors were gathered, and the efficacy and mechanism of CAM therapy were analyzed.

### 2.1. Chinese Herbal Medicine

CHM can increase the reproductive capacity of infertile patients in various ways, whose efficacy and safety in treatment have attracted growing attention and recognition [24, 25]. A Korean researcher pointed out that a growing number of infertile couples would choose CHM to treat infertility [26]. CHM can restore the endometrial environment to normal and provide fertile “soil” for zygote by improving endometrial blood circulation, increasing the level of sex hormones in the uterus, promoting endometrial thickening, and affecting the morphology of the uterus [27, 28], thus achieving the goal of treating infertility and normalizing pregnancy.

#### 2.1.1. The Application of Chinese Herbal Medicine in Clinical Practice

In many clinical trials, CHM has been proved to be effective in the treatment of infertility caused by endometrial lesions such as thin endometrium, low endometrial receptivity, adenomyosis, and endometritis.

To investigate the clinical efficacy of Gushen Antai pill (GAP; kidney-tonifying and miscarriage-preventing pill) in the treatment of infertility due to thin endometrium, Wu et al. [29] selected 83 patients with thin endometrial infertility and randomly divided them into two groups, observing the endometrial thickness on the day of mature follicles. The results showed that though the endometrial thickness of the two groups was increased after treatment, the outcome of endometrial thickening in the GAP group was better than that of the control group (*P* < 0.05). Yin et al. [30] divided 60 infertile patients with thin endometrium who had no significant differences in age, weight, duration of infertility, mid-luteal endometrial thickness, and sex hormone levels into two groups to observe the effect of Bushen Huoxue recipe (kidney-tonifying and blood-promoting recipe) on the endometrium. The results showed that the pregnancy rate of the observation group was 43.3%, which was significantly higher than that of the control group (*P* < 0.05), and the endometrial thickness increased from 6.2 ± 2.1 mm to 9.6 ± 1.0 mm, which was significantly higher than that of the control group (*P* < 0.05). Moreover, on the basis of conventional Western medicine, Bushen Yanggong decoction (kidney-tonifying and uterus-nourishing decoction), Yangjing Zhongyu decoction (kidney essence-nourishing and pregnancy-helping decoction), Jinfeng pill (Golden Phoenix Pill), and Bubao decoction (uterus-nourishing decoction) can be used to treat thin endometrial infertility, increase endometrial thickness, and improve pregnancy rate [31–34]. CHM, which has the function of supplementing the kidney, activating blood, and filling essence, can effectively improve the operation and distribution of essence, qi, blood, and body fluid in the uterus, so as to give the endometrium power to grow, which is similar to the promotion of endometrial growth by modern medicine.

In addition to increasing endometrial thickness, CHM can effectively improve endometrial receptivity of infertile patients via various ways [35, 36]. To explore the effect of Danyu decoction (DYD; salvia and Evodia decoction) on endometrial receptivity of infertile patients, Liu et al. [37] randomly divided 50 patients into two groups: the observation group was treated with DYD, while the control group was treated with vitamin E. The final results showed that DYD could promote the endometrium transform to type A, which is easy to conceive and reduce the uterine artery resistance index (RI) and pulsatility index (PI), so as to get better blood perfusion for uterus. The statistical results of pregnancy showed that the pregnancy rate of the observation group was significantly higher than that of the control group (*P* < 0.05). Through clinical observation, Geng et al. [38] concluded that Jieyuyubao pills (JYP; stagnation-relieving and uterus-nourishing pill) could increase the rate of type A endometrium, effectively improve endometrial receptivity, and promote the implantation of zygote, thereby increasing the pregnancy rate. Meanwhile, JYP could also significantly reduce the liver depression symptoms including chest distension, mental depression, and irritability, and lower the syndrome score. Fang et al. [39] designed a randomized, double-blind, and placebo-controlled trial to investigate the effect of Erzhi Tiangui granule on DNA methyltransferase (DNMT) expression in the endometrium of infertile patients with kidney yin deficiency. The experimental results showed that the expression of DNMT1 in the endometrium of the treated group was significantly higher than that of the placebo control group; besides, the endometrium showed a typical decidual endometrium with normal growth of mesenchyme and glands and normal secretion of glandular epithelium. CHM with kidney-tonifying effect such as Yulin Zhuyun prescription (helping pregnancy and giving birth to fetus prescription) and Kuntai capsule (women's health-promoting pill) can promote the expression of integrin *αvβ*3 and GLUT4 on endometrium [40, 41]. As indicators to evaluate endometrial receptivity, the better the endometrial receptivity, the higher the levels of integrin *αvβ*3, GLUT4, and DNMT expression. The high expression of these proteins in the endometrium of patients after treatment also indirectly demonstrated the improvement of endometrial receptivity by CHM [42–44]. Xu et al. [45] performed biopsies on the endometrium in 72 patients with infertility due to endometrial lesions and found that the endometrial pinopodes of infertile patients who treated with Wenjing Quyu prescription (essence-warming and blood stasis-removing prescription) were richer in structures, and the area of the endometrial surface covered with pinopodes was more than that of the control group. It is concluded that CHM can effectively increase the growth of pinopodes in the mature stage of endometrium, thus improving endometrial receptivity [46].

Infertility caused by adenomyosis is mostly related to the changes in uterine cavity shape, decreased endometrial receptivity, and local or overall chronic inflammatory response [47, 48]. The treatment of adenomyosis in CHM is mostly based on the direction of qi-blood dialectic, and the methods such as boosting qi, invigorating blood, and dispelling stasis have been widely applied, which can effectively relieve the symptoms of adenomyosis, control local lesions, and improve the pregnancy rate [49]. Shaoyao-Gancao decoction (Paeonia and Licorice decoction), as a classic ancient recipe, is often used to treat gynecological diseases. Clinical experiments have confirmed that both Shaoyao-Gancao decoction and Jiawei Shaoyao-Gancao decoction (supplemented Paeonia and Licorice decoction) can inhibit the migration, proliferation, and differentiation of endometrial cells, prevent the occurrence of adenomyosis, and provide a good intrauterine environment for pregnancy [50, 51]. Pan et al. [52] randomly divided 60 patients with infertility due to adenomyosis into two groups: the control group was treated with urinary follicle-stimulating hormone + chorionic gonadotropin + letrozole, while the observation group was treated with Huayu Xiaozheng prescription (HXP; blood stasis-removing and abdominal mass-resolving prescription) on this basis. After 3 menstrual cycles, the intrauterine spontaneous pregnancy rate in the observation group reached 60%. The uterine volume, endometrial thickness, PI, and RI in the observation group were significantly better than those in the control group (*P* < 0.05). The results suggested that HXP could effectively slow down the rate of uterine enlargement, increase the thickness of endometrium, improve the blood flow of intrauterine artery, and increase the intrauterine pregnancy rate of patients. In addition, Sanjie Zhentong capsule (static blood-resolving and pain-alleviating capsule), Yishen Sanjie Quyu decoction (kidney-invigorating and static blood-resolving decoction), and Wenjing Huoxue decoction (essence-warming and blood-promoting decoction), all of which can improve pregnancy rate and treat infertility caused by adenomyosis via improving TCM syndromes, controlling uterine volume, and helping the recovery of uterine artery blood flow [53–55].

Endometritis means the inflammatory state of the endometrium, which is associated with adverse reproductive outcomes. This status is able to negatively affect the endometrial receptivity and act on detriment to embryonic implantation, thus becoming one of the causes of female infertility [56, 57]. Zhou et al. [58] performed hysteroscopic endometrial biopsies in 58 infertile patients caused by asymptomatic endometritis; they found that the endometrium in the Penning granule (PNG; peaceful pelvic cavity granules) treatment group basically restored the endometrium to normal, vascular proliferation disorder, endometrial edema, and punctate or scattered hemorrhage almost disappeared, the endometrial interstitial lymphocyte infiltration was significantly reduced, and the expression of immunohistochemical index CD38 for the diagnosis of endometritis and the expression of inflammatory factor HIF-*α* were also significantly decreased. The overall pregnancy rate of 54.55% in the treatment group was significantly higher than that of the control group (*P* < 0.05). The results suggested that PNG could eliminate endometrial inflammation, promote the improvement of endometrial receptivity, and increase the clinical pregnancy rate.

Through searching and reading the relevant literature, we found that the clinical reports of CHM for the treatment of infertility caused by endometrial factors were mainly from Chinese medical institutions, while the related research in Western medical institutions mostly focuses on the mechanism research based on animal experiments, whereas much attention has not paid to clinical research. What's more, the sample size of various clinical studies in China is small and single; most of the clinical studies only focus on the evaluation of clinical efficacy, while less attention has been paid to the study of mechanism (see Table 1).

#### 2.1.2. Therapeutic Mechanisms of Chinese Herbal Medicine

The mechanisms of CHM in the treatment of infertility caused by endometrial factors are relatively complex. The possible mechanisms are summarized in the following four points.

#### 2.1.3. Regulation of Endocrine and Promotion of Endometrial Development

Estrogen and progesterone and their receptors play a key role in the proliferation and transformation of the endometrium and embryo implantation. CHM can promote endometrial development and improve endometrial receptivity by regulating the content of endometrial estrogen and progesterone and the expression of their receptors, providing a good microenvironment for embryo implantation and increasing the success rate of pregnancy in infertile patients [59].

Huang et al. [60] explored the effect of Dingkun pill (DK; women's health-stabling pill) on endometrial receptivity and implantation in mouse by establishing different mouse models, and concluded that DK could promote embryo implantation by promoting the expression of estrogen target genes leukemia inhibitory factor (LIF), lactotransferrin (Ltf), insulin growth factor-1 (IGF-1), and spondin 2 (Spon2), altering the uterine responsiveness to estrogen. In the delayed implantation mouse model, DK could establish endometrial receptivity under the premise of suboptimal endometrial estrogen content and improve embryo implantation rate. The experimental results fully confirmed the value of DK in infertility treatment, especially in infertile patients with poor endometrial response to hormones. Tian et al. [61] studied the effect of Ankun Zhongzi Wan (AZW; women-stabling and pregnancy-promoting pill) on the expression of estrogen receptor (ER) and progesterone receptor (PR) in mice with embryonic implantation disorders. And the experimental results showed that ER and PR were strongly expressed in endometrial epithelial and mesenchymal cells in the AZW group, which were better than the model group (*P* < 0.05). The pregnancy rate, average number of embryos born, and embryo implantation rate in the AZW group were also significantly higher than those in the model group (*P* < 0.05). The experimenters believed that AZW could significantly improve the expression of ER and PR in the endometrium of female mice, promote the implantation and development of blastocysts, and improve the pregnancy rate. In addition, there are other CHM with the functions of tonifying the kidney, invigorating the blood, and boosting the qi, such as Bushen Yiqi Huoxue recipe (kidney-tonifying, qi-benefiting and blood-promoting recipe), Tiaojing Zhuyun capsule (menstruation-regulating and pregnancy-promoting capsule), and Zhuyun I recipe (pregnancy-promoting I recipe), all of which can increase the serum estrogen and progesterone levels in mice with impaired implantation, enhance the effect of estrogen and progesterone on endometrium, improve the expression of ER and PR, promote the maturation of the endometrium, and provide good conditions for pregnancy [62–64].

#### 2.1.4. Improvement of Endometrial Thickness, Blood Flow, Pinopodes, and Other Endometrial Morphology

TCM holds the belief that “blood is the precondition for women” and emphasizes the application of “blood regulation” in the treatment. CHM can treat infertility caused by endometrial abnormalities from multiple angles and levels by improving blood flow, nourishing the endometrium, and promoting endometrial thickening [65].

In order to investigate the effect of Bushen Tiaoxue granules (BTG; kidney-tonifying and blood-promoting granules) and Kunling Wan (KW; female Poria cocos pill) treatment on vascular function and endometrial receptivity in mice and its mechanism, Lv et al. [66] conducted animal experiments and concluded that BTG and KW could improve endometrial angiogenesis, increase endometrial receptivity, and improve pregnancy rate. The main mechanism of action was that BTG and KW could increase the content of blood collaterals in the uterus, increase blood flow, upregulate the expression of vascular endothelial growth factor A (VEGFA) and CD31 in the endometrium, and promote the increase in the number of mature vessels and neovascularization. It also increased the thickness and area of the endometrium, and improved the number, density, and secretory activity of endometrial glands. Several investigators found that CHM such as Bushen Huoxue recipe (kidney-nourishing and blood-promoting recipe) and Xiaoyao powder (Free Wanderer Powder) could increase the expression of endometrial pro-angiogenic factors such as HIF1*α* and P38, the signaling pathways of COX-PGE2, JNK MAPK, and PI3K/Akt/eNOS, and the expression of its downstream factors such as MMP2, MMP9, PCNA, and FGF2, and then promote its receptor-mediated endometrial angiogenesis, improve endometrial receptivity, and promote embryo implantation, thereby treating infertility [67, 68]. In addition, some formulas such as Tiaojing Cuyun recipe (regulating menstruation and helping pregnancy recipe) and Wenshen Yangxue decoction (kidney-warming and blood-nourishing decoction) have been shown in animal experiments that they can not only promote the formation of endometrial blood vessels, but also increase the number of well-developed mature pinopodes in the endometrium [69, 70]. Yu et al. [71] used pinopodes as an important morphological indicator of endometrial receptivity to further explore the mechanism of Zhuyun recipe (ZR; pregnancy-promoting recipe) in the treatment of infertility in mice with embryo implantation disorders. The results showed that pinopodes were less in the mouse model group with embryo implantation disorders, and there were only a few microvilli on the surface, which were poorly developed. However in the ZR group, the number of pinopodes increased compared with that in the model group, with prominent shape, clear boundary, and complete development. Therefore, ZR could improve the endometrial environment of mice with embryo implantation disorders and increase the pregnancy rate by improving the morphology of the pinopodes.

#### 2.1.5. Modulation of the Expression of Related Factors and Improvement of Endometrial Receptivity

The normal endometrium allows zygote to implant only during a specific brief period called the “implantation window” [72]. During this period, some molecular markers such as integrin *αvβ*3, LIF, and osteopontin (OPN) all play important roles. They have a cycle-specific expression pattern, which are upregulated and highly expressed during the period, and are able to mediate adhesion, migration, and signal transduction between cells, participate in the decidual process of the endometrium, and promote embryo implantation [73–75]. By promoting the expression of these related factors, CHM can improve the receptivity of the endometrium, increase the probability of embryo implantation, and avoid the occurrence of pregnancy failure [76].

Li et al. [77] studied the expression of integrin *αvβ*3 in rat endometrium during implantation period and the effect of Yiqixue Buganshen recipe (qi- and blood-tonifying, liver and kidney-nourishing recipe) on the expression of it. They found that the expression of integrin *αvβ*3 in rat endometrium was time-dependent and was closely related to the gestation process. Compared with the low expression in the model group, the expression of integrin *αvβ*3 in the treatment group was significantly increased (*P* < 0.01). Terawaki believed that LIF played an indispensable role in embryo implantation. According to animal experiments, he found that Tokishakuyakusan, a kind of Japanese Kampo commonly used to treat infertility in Japan, could significantly improve the production of LIF protein and the expression of LIF mRNA in endometrial cells and reduce the decidual dysfunction in rats with implantation disorders, resulting in improving embryo implantation conditions, thus treating infertility [78]. In addition, Zishen Yutai pill (kidney-nourishing and fetus-raising pill), modified Shoutaiwai recipe (modified ex-longevity fetus recipe), and Bushen Huoxue recipe (kidney-nourishing and blood-promoting recipe) have all been proved by animal experiments to upregulate the expression of integrin *αvβ*3 and LIF on endometrium to increase endometrial receptivity and embryo implantation rate [79–81].

#### 2.1.6. Inhibition of Inflammatory Response and Restoration of Normal Uterine Environment

Inflammation plays an important role in endometrial diseases, and adenomyosis, endometritis, and other diseases are all related to it [82]. The inflammatory response of the endometrium leads to the disruption of the endometrial microenvironment, impairment of the embryonic implantation process, and poor placental formation, which ultimately leads to infertility [83].

According to animal experiments, Ying et al. [84] found that Qiu's Neiyi recipe could inhibit the activated MAPK signaling pathway on the endometrium and reduce the expression of inflammatory factors in uterine tissues, thus effectively reducing the inflammatory response in the endometrium. Jing et al. [85] studied the changes in the expression of NF-*κ*B and TGF-*β*1 in the endometrium of rats with chronic endometritis after the treatment with modified Danggui Shaoyao powder (MDSP; Modified Chinese Angelica and Peony Powder). They found that MDSP could inhibit the endometrial inflammatory response by downregulating the expression levels of NF-*κ*B and TGF-*β*1 proteins and effectively improve the endometrial receptivity in rats with chronic endometritis. Er-Miao-Fang has been confirmed to exert anti-inflammatory effects by inhibiting NF-*κ*B and MAPK signaling pathway proteins [86]. Moreover, Shaofu Zhuyu decoction (lower abdominal stasis-expelling decoction) has been confirmed to reduce the levels of oxidative stress indicators ROS, malondialdehyde, and inflammatory factors including TNF-*α*, IL-6, and IL-8, thereby reducing endometrial oxidative stress and inflammatory response in the endometrium [87]. Bushen Tiaochong decoction (kidney-nourishing and Chong channel-harmonizing decoction) has been shown to downregulate the expression of the apoptosis-related factors including caspase-1, IL-1*β*, and IL-18, and reduce the inflammatory response of the damaged endometrium [88]. Accordingly, it can be seen that CHM plays an important role in treating endometrial inflammation and providing a favorable endometrial environment for embryo implantation.

In general, the mechanism of CHM in the treatment of infertility caused by endometrial factors is rather complicated. In order to provide a better theoretical basis for clinical work, more basic experimental studies are needed to systematically and elaborately clarify the therapeutic mechanism of CHM (see Table 2).

### 2.2. Acupuncture and Moxibustion Therapy

Acupuncture and moxibustion therapy originated from the “The Yellow Emperor's Inner Classic,” having a history of more than 2,000 years. It is guided by the acupoint theory, using filiform needles and mugwort as materials and tools to achieve the purpose of preventing and treating diseases mainly by inserting thin sterile metal needles into specific areas of the body or burning cauterizing mugwort to stimulate certain parts of the body, accompanied by certain therapeutic techniques [89–91].

A retrospective analysis of clinical trials has shown that filiform acupuncture is the most commonly used method in the field of ART, and it is most widely used in clinical practice due to its simplicity and efficiency. Additionally, acupuncture combined with moxibustion and electric acupuncture (EA) are also commonly used methods [92]. As an important branch of CAM, with fewer side effects and higher acceptance, acupuncture has attracted the attention of many clinical practitioners; thus, a series of clinical and basic research has been carried out.

#### 2.2.1. The Application of Acupuncture in Clinical Practice

Acupuncture, as one of the external treatments in TCM, can not only prevent the occurrence of diseases, but also be used as a complementary and alternative treatment after the occurrence of diseases [93]. The history of acupuncture treating infertility can be traced back to 1999. That was the first time researchers found that acupuncture could significantly increase fertility rate, pregnancy rate, and the number of live births [94]. Subsequently, in 2002, Paulus et al. [95] showed that acupuncture could significantly increase the pregnancy rate of patients. As a TCM treatment, acupuncture has the unique advantages of convenience, safety, effectiveness, rapidity, and cheapness, making it attract the attention of many domestic and foreign scholars in the field of reproductive medicine [96]. The normal implantation of embryos is closely related to the endometrium [74]. A meta-analysis showed that acupuncture therapy could safely and effectively improve endometrial receptivity by increasing endometrial thickness, improving endometrial morphology, and improving uterine blood circulation. In addition, acupuncture can also relieve tension and anxiety of infertile patients, thus improving their quality of life and pregnancy outcome [97].

#### 2.2.2. The Curative Effect of Acupuncture

In recent years, acupuncture, as a simple and easy-to-operate CAM, has been gradually accepted by most infertile patients, and its positive effects on endometrium have also been confirmed in many studies. Quantities of studies have shown that acupuncture alone or combined with other treatments is more effective than nonacupuncture in improving endometrial receptivity and increasing endometrial thickness [98–100].

Li et al. [101] selected 60 patients with thin endometrial infertility and randomly divided into the treatment group and the control group, 30 cases in each group. The treatment group was given acupuncture combined with CHM treatment, and the control group was given Western medicine treatment. The results showed that the endometrium in the treatment group was obviously thickening than that in the control group, and the pregnancy rate of the treatment group was also significantly higher than that of the control group (*P* < 0.05). This experiment showed that acupuncture with CHM could effectively treat infertility caused by thin endometrium, promote endometrial growth, improve endometrial receptivity, and increase the clinical pregnancy rate of patients with thin endometrial infertility. In order to observe the effect of acupuncture on the endometrium and pregnancy outcome in patients with ovulatory disorders, Xu et al. [102] randomly divided 60 infertile patients into two groups. The control group received conventional ovulation induction program, and the treatment group was treated with acupoints such as Baihui (DU20), Mingmen (DU4), Geshu (BL17), Guanyuan (RN4), and Qihai (RN6) on the basis of the treatment of the control group. The results showed that the embryo implantation rate and clinical pregnancy rate of patients in the treatment group were higher than those in the control group (*P* < 0.05), indicating that acupuncture could improve their endometrial receptivity, and increase the embryo implantation rate and clinical pregnancy rate of infertile patients based on the conventional ovulation promotion protocol. Through clinical observation of 60 infertile patients, Wang et al. [103] concluded that acupuncture treatment could effectively reduce the PI and RI of patients, increase endometrial blood perfusion, improve endometrial receptivity, and significantly increase the pregnancy rate. Wang et al. [104] also found that the combination of acupuncture and medicine treatment could significantly increase endometrial thickness, improve uterine blood circulation, and help to improve endometrial receptivity.

In order to observe the clinical efficacy of acupuncture and moxibustion combined with umbilical application with TCM in thin endometrial infertility, Yang et al. [105] randomly divided 126 patients with thin endometrial infertility into a treatment group and a control group: the control group was treated with Western medicine, and the treatment group was treated with acupuncture and moxibustion combined with umbilical application with TCM on the basis of the control group. The acupoints were selected from Guanyuan (RN4), Taixi (KI3), Sanyinjiao (SP6), Shenshu (BL23), Mingmen (DU4), Taichong (LV3), and Xingjian (LV2). The results showed that endometrial thickness, pregnancy rate, and live birth rate in the treatment group were significantly higher than those in the control group (*P* < 0.05). Xu et al. [106] concluded that staged acupuncture with moxibustion treatment could also increase endometrial thickness and promote endometrial growth, thus improving the clinical pregnancy rate.

Studies have confirmed that acupuncture using “Tongyuan acupuncture” at specific acupoints can increase endometrial thickness, improve endometrial receptivity, and improve pregnancy outcome in patients with repeated implantation failure (RIF) of thin endometrium [107]. Xue et al. [108] randomly divided 74 patients with RIF of thin endometrium that are to be underwent frozen-thawed embryo transfer into treatment group and control group. The control group was given oral Western medicine, and the observation group was given acupuncture treatment based on the Tongyuan acupuncture method on the basis of the control group. Acupoints such as Baihui (DU20), Dazhui (DU14), Qihai (RN6), and Guanyuan (RN4) were selected. After treatment, the clinical pregnancy rate of the treated group was 37.8%, which was higher than the 16.2% in the control group, and the endometrial thickness in the treated group was also higher than that in the control group.

Although a large number of clinical trials have demonstrated the efficacy of acupuncture as a complementary and alternative therapy in the treatment of infertility caused by endometrial factors, most of the clinical experiments have small sample sizes and there is variability in acupuncture manipulation and acupoint selection. Therefore, it is necessary to further expand the sample size, deeply explore the relevant mechanisms of action, and formulate reasonable clinical protocols (see Table 3).

#### 2.2.3. Therapeutic Mechanisms of Acupuncture

Scientific studies have concluded that the insertion of acupuncture into the skin creates a holistic connection with the nervous system, immune system, and endocrine system through meridians [113]. A great deal of studies have shown that the mechanism of acupuncture-assisted treatment for infertility induced by endometrial factors may be related to three aspects of local microcirculation in the uterus, reproductive endocrine, and molecular biology.

#### 2.2.4. Improvement of Endometrial Microcirculation

Studies have shown that acupuncture can inhibit the activity of the central sympathetic nerve, reduce the uterine blood flow resistance, promote local blood circulation and the development of the endometrium, and improve the receptivity of the endometrium by regulating the hypothalamus-pituitary-ovarian (HPO) axis, which has positive significance for embryo implantation and ultimately improves the clinical pregnancy rate and live birth rate [114].

In a clinical trial [109], 120 infertility patients were randomly divided into three groups: acupuncture test group, acupuncture control group, and blank control group. In the acupuncture group, Guanyuan (RN4), Zhongji (RN3), Zigong (EX-CA1), Sanyinjiao (SP6), Guilai (ST29), and Xuehai (SP10) were selected. In the control group, acupoints were selected from Fengshi (GB31), Yanglingquan (GB34),Waiguan (SJ5), and Sidu (SJ9). And the blank control group was treated with nonacupuncture. The results showed that the spiral artery PI, RI, and *S*/*D* values in the pregnancy group were significantly lower than those in the nonpregnancy group, and the spiral artery PI, RI, and *S*/*D* values in the acupuncture group were lower than those in the blank control group, with a statistically significant difference (*P* < 0.05). Acupuncture can also stimulate the dopamine system in the brain, regulate the entire reproductive system, promote blood circulation in the uterine arteries, and improve endometrial receptivity [115]. Therefore, it is believed that acupuncture can improve endometrial microcirculation mainly by decreasing the uterine spiral artery blood flow index, improving endometrial blood circulation, and increasing endometrial perfusion, thereby increasing endometrial thickness and endometrial tolerance, which increases pregnancy rate in turn [116, 117].

#### 2.2.5. Regulation of Reproductive Endocrine

From the aspect of reproductive endocrine, the endometrium is the target organ of estrogen and progesterone. Sufficient estrogen and progesterone is one important part of endometrium to complete conception, and the functions of estrogen and progesterone are closely related to the expression of their receptors in turn. Liu et al. [118] randomly divided rats into the normal group, the model group, and the acupuncture group in order to observe the effect of acupuncture on embryo implantation in rats with embryo implantation disorders and preliminarily explore its mechanism of action. Acupuncture was performed at acupoints of “Zusanli (ST36),” “Sanyinjiao (SP6),” and “Taichong (LV3).” And the serum levels of estradiol, progesterone, and prolactin, and the expression of PR and prolactin receptors at implantation site were all detected. The results showed that the acupuncture group could significantly increase the serum levels of estradiol and progesterone, as well as the expression of PR on the endometrium, and the implantation rate and the average number of implanted embryos in the acupuncture group were significantly higher than those in the model group (*P* < 0.01), which may be related to acupuncture stimulation that could enrich the expression of ER and PR on the endometrium of rats with embryo implantation disorders at the same time and exert physiological effects on the endometrium.

#### 2.2.6. Regulation of the Expression of Related Proteins and Factors

The normal implantation of zygote is related to specific molecular markers on the endometrial surface [119]. Current studies have found that integrins, VEGF, etc., can be considered as markers of endometrial receptivity [120], and the regulatory effect of endometrium by molecular markers depends on the “hypothalamus-pituitary-ovarian-uterine” reproductive axis. Moreover, studies have also shown that acupuncture can promote the expression of molecular markers by modulating this reproductive axis, thus improving endometrial receptivity and promoting the growth and development of endometrium [121, 122].

Integrins are a class of cell adhesion molecules that widely exist in endometrium, which can be divided into 3 subtypes: *α*1*β*1, *α*4*β*1, and *αvβ*3. The establishment of high endometrium receptivity is based on the simultaneous expression of the three subtypes [122, 123]. Zhang et al. [124] observed the effect of acupuncture on the implantation of blastocysts in rats and found that compared with the clomiphene group, rats in the clomiphene-combined acupuncture group had better endometrium development, and the embryo implantation rate was significantly higher than that of the model group. The investigators suggested that acupuncture may significantly improve the poor endometrial receptivity status caused by clomiphene ovulation treatment by regulating the protein integrin *αvβ*3 and its mRNA expression, a marker molecule protein of endometrial receptivity. VEGF has the ability to stimulate the proliferation and differentiation of endometrial cells, and can affect local angiogenesis in the endometrium directly. He et al. [125] randomly divided early pregnant rats into the normal group (N), model group (M), acupuncture group (A), and nonacupuncture group (C), and acupuncture points of “Zusanli (ST36)” and “Sanyinjiao (SP6)” were taken in the acupuncture group. The results showed that the pregnancy rate and the average number of implanted embryos in group A were significantly higher than those in groups M and C (*P* < 0.05), which may be related to the increase in VEGF expression in the uterus of rats with embryo implantation disorders by acupuncture.

Moreover, a study performed microRNA sequencing on endometrial samples from infertile women who had received acupuncture or not, and then compared the differences in the two DEmiRNAs and predicted their functions. The results showed that DEmiRNAs may be involved in acupuncture treatment through endocytosis, axon guidance, oxytocin signaling pathway, hippopotamus signaling pathway, and estrogen signaling pathway. And hsa-miR-449a, hsa-miR-3135b, hsa-miR-345-3p, and their target genes were also constructed with miRNA-gene network, which jointly affected endometrial receptivity [126]. Additionally, some researchers also conducted high-throughput RNA sequencing and bioinformatics analysis on the samples of patients who had received acupuncture treatment or not, and concluded that acupuncture treatment could play a role in changing endometrial receptivity by regulating the differential expression of circular RNAs in infertility patients [127]. Yuan et al. [128] performed Erbuzhuyu decoction (EBZYD; two-step Evodia decoction) combined with acupuncture on mice, and the results showed that it could promote the expression of endometrial tolerance-related factors and increase blastocyst number and endometrial thickness through activating PI3K/Akt/mTOR signaling pathway, and its treatment effect was superior to using EBZYD or acupuncture alone (see Table 4).

#### 2.2.7. The Application of Electroacupuncture in Clinical Practice

EA is a therapy that applies electric stimulation to the needle to enhance the stimulation effect during the retention of acupuncture after acupoints have received qi. Previous studies have shown that EA stimulation of acupoints such as Zhongji (RN3), Guanyuan (RN4), Sanyinjiao (SP6), and Zigong (EX-CA1) can not only improve endometrial blood flow and endometrial receptivity, but also improve female reproductive capacity by regulating neuroendocrine and immunity [135].

#### 2.2.8. The Curative Effect of Electroacupuncture

Zhong et al. [110] performed EA intervention on in vitro fertilization-embryo transfer (IVF-ET) patients with kidney deficiency and phlegm stasis, and found that EA could improve endometrial blood flow and increase endometrial receptivity, which positively affected pregnancy outcome. Yu et al. [111] randomly divided 80 PCOS patients into EA combined with the Western medicine group and the Western medicine group. In the combined group, Qihai (RN6), Guanyuan (RN4), Zigong (EX-CA1), Dahe (KI12), Sanyinjiao (SP6), Zhongji (RN3), Diji (SP8), Shenshu (BL23), Sanjiao Yu (BL22), and Ciliao (BL32) were used as the main acupoints. The results showed that the endometrial thickness and the rate of type A endometrium were better in the combined group than those in the Western medicine group after treatment. In addition, the serum estrogen and progesterone levels of the patients after treatment were significantly higher than those before treatment. Therefore, it was concluded that the combined EA treatment could not only thicken the endometrium and improve its morphology, but also significantly increase the serum levels of estrogen and progesterone, which played a role in improving the endometrial receptivity.

He et al. [112] randomly divided 80 patients with ovulatory dysfunction infertility into the observation group and the control group with 40 patients in each group. The observation group received EA treatment at Guanyuan (RN4), Zhongji (RN3), Zigong (EX-CA1), Qihai (RN6), Sanyinjiao (SP6), and other acupoints until HCG day, while the control group was given aspirin tablets orally from the 7th day of the menstrual cycle until HCG day. The experimental results indicated that the endometrial receptivity-related indicators and pregnancy rate of the observation group were significantly better than those of the control group. Kong et al. [136] performed early intervention of EA on 310 patients, and selected Guanyuan (RN4), Zigong (EX-CA1), Sanyinjiao (SP6), and Taixi (KI3) acupoints for sparse and dense wave therapy. The results showed that endometrial thickness and pregnancy rate all increased, and the curative effect was relatively significant (see Table 3).

#### 2.2.9. Therapeutic Mechanisms of Electroacupuncture

EA has some effects on the endometrium in terms of reproductive endocrinology, genetics, and molecular biology likewise. It can promote the formation of the pineal gland and enhance endometrial receptivity in the thin endometrium model rat through multiple molecular targets [137]. Therefore, infertile patients with thin endometrium can receive complementary and replacement therapy by EA to increase the number of blood vessels and glands in the endometrium, and ultimately achieve the purpose of improving the shape of the endometrium.

Firstly, EA can promote the proliferation of thin endometrial cells and elevate the levels of ER and PR. Meng et al. [129] divided SD rats into a blank group, a model group, a cell group, and a combined group, with 10 rats in each group. The combined group was given EA on “Guanyuan (RN4),” “Zigong (EX-CA1),” and “Sanyinjiao (SP6).” It was found that the uterine coefficient and the expression of ER and PR in the cell group and the combined group were significantly increased compared with those in the model group, and the expression of PR in the combined group was higher than that in the cell group (*P* < 0.05). According to years of experience in diagnosis and treatment, Dr. Liu often uses low-frequency stimulation during treatment, based on the fact that the main induction site of low-frequency stimulation is in the hypothalamus, which can bidirectionally regulate the function of the HPO axis, thereby increasing the secretion of estrogen, balancing the dynamic relationship between follicle-stimulating hormone and luteinizing hormone, promoting follicle maturation and the release of dominant follicles, improving ovarian reserve function and endometrial receptivity, increasing pregnancy success rate, and improving pregnancy outcome [138].

Secondly, EA can improve endometrial receptivity by affecting the expression of related factors. It was found that the expression of homologous frame gene 10 (HOXA-10) was closely related to endometrial tolerance; namely, a low level of HOXA-10 expression was indicative of low endometrial tolerance [139]. Jiang et al. [130] found that EA combined with Bushen Huoxue recipe could improve the endometrial receptivity by increasing the expression of HOXA-10 on the endometrium through the observation of PCOS rats after ovulation induction. Xi et al. [131] evaluated endometrial regeneration and endometrial receptivity in thin endometrium rats treated with EA, then concluded that EA could increase the formation of pinopodes through multiple molecular targets, improve endometrial receptivity, significantly increase embryo implantation rate, and improve pregnancy outcome. In addition, You et al. [132] suggested that high-frequency EA could enhance endometrial receptivity and promote embryo implantation in ovarian hyperstimulation model rats by enhancing the expression of the LIF/STAT3 signaling pathway. As molecular markers on the surface of the endometrium, IGF-1 and LIF, act on mediating embryo implantation and influencing endometrial morphology and receptivity [140]. Lin et al. [133] found that EA treatment could upregulate the expression of IGF-1 protein in the endometrium of female mice, significantly increase the average number of implantation sites, and improve the pregnancy rate (*P* < 0.05). Fu et al. [141] found that LIF expression was elevated in mice treated with EA, the growth of endometrial glands was promoted, and the pregnancy rate was also improved.

Generally, both conventional acupuncture treatment and EA can effectively treat infertility induced by endometrial factors. In clinical treatment, they have been accepted and recognized by a growing number of doctors and patients. Nevertheless, there is still a need for more clinical and basic experiments to explore more precise curative effects and mechanisms to provide a solid theoretical basis for the better application of acupuncture in the clinical treatment of infertility (see Table 4).

#### 2.2.10. The Application of Moxibustion Therapy in Clinical Practice

Moxibustion therapy refers to a treatment that is commonly applied in clinical practice, using the generated moxa heat mainly by burning the moxa sticks or moxa columns made of moxa leaves to stimulate acupoints or specific parts directly or indirectly, which has a certain effect on diseases with cold pathogens as the main point of syndrome differentiation [142]. It achieves the purpose of disease prevention and treatment by stimulating meridian qi and regulating physiological and biochemical functions of the human body, whose mechanism is similar to that of acupuncture, exists side by side, and plays a part together. Moxibustion has many advantages, such as simple operation, low cost, and remarkable effect. Among them, warm moxibustion, wheat grain moxibustion, medicinal moxibustion, and thunder fire moxibustion are commonly used to improve the endometrium and increase the pregnancy rate in clinical practice.

#### 2.2.11. The Curative Effect of Moxibustion Therapy

*(1) Warm Acupuncture.* Warm acupuncture is to transmit the heat generated by the burning of the moxa columns to the deep tissue continuously through the needle body on the basis of needle piercing to get qi, which promotes circulation of organs and tissues smoothly and accelerates metabolism. Warm acupuncture can improve the poor endometrial receptivity status to some extent [143]. Liang and Mo [144] selected 92 patients with thin endometrial infertility and divided them into two groups: 46 patients in the control group were treated with Western medicine, while 46 patients in the observation group were treated with warm acupuncture, and their clinical effects were analyzed. The results showed that the curative effect indicators such as endometrial thickness, endometrial type, pregnancy rate, and endometrial blood flow in the observation group were better than those in the control group after treatment, indicating that warm acupuncture could improve the endometrial receptivity and the clinical pregnancy rate effectively in the treatment of infertile patients secondary to thin endometrium. In order to explore the efficacy of treating endometrial infertility with kidney deficiency and blood stasis, Li [145] divided 136 patients who met the inclusion criteria into two groups randomly: the control group was treated with estradiol valerate orally, while the observation group was treated with warm acupuncture combined with Zhenqi decoction on the basis of the treatment of the control group. Then, the endometrial thickness of the two groups was detected. The results showed that the endometrial thickness in the observation group was higher than that in the control group, which further confirmed that warm acupuncture combined with Zhenqi decoction (glossy fruit and wolfberry decoction) could improve the endometrial receptivity, thus increasing the pregnancy rate. Luo et al. [146] used warm acupuncture for pretreatment in order to improve the endometrial receptivity of frozen embryo transfer patients, which found that warm acupuncture may improve these patients' receptivity of endometrium by improving the endometrial morphology and blood flow, improve the embryo implantation rate and clinical pregnancy rate, and reduce the early miscarriage rate. Su et al. [147] studied the changes of endometrial receptivity of IVF-ET failures who received acupuncture, EA, and warm acupuncture. In the acupuncture group, common acupuncture treatment was applied at the follicular stage after menstruation and stopped at ovulation stage. The EA group was treated with EA on the basis of the acupuncture group. The warm acupuncture group was treated with warm acupuncture on the basis of the acupuncture group. After continuous treatment for 3 menstrual cycles, it was found that the rate of type A endometrium, subendometrial flow type A rate, embryo implantation rate, and clinical pregnancy rate in the warm acupuncture group were significantly higher than those in the other two groups, indicating that warm acupuncture could improve endometrial receptivity of IVF-ET failures more effectively, thereby increasing the success rate of embryo transfer.

*(2) Thunder Fire Moxibustion.* It is a kind of the moxibustion method that uses moxa and various medicines to compose plant medicine columns according to a certain proportion, which is based on modern anatomy for syndrome differentiation and treatment and supplemented by acupoints. As an integral part of moxibustion, thunder fire moxibustion has been proved to be effective in treating infertility caused by endometrial factors in many clinical experiments.

Thunder fire moxibustion can improve the pregnancy rate of infertile patients with adenomyosis safely and effectively. Chen et al. [148] selected 60 patients with adenomyosis combined with infertility and TCM syndrome differentiation belonged to the diagnosis of cold congealing and blood stasis, and divided them into the treatment group and the control group randomly, with 30 cases in each group. The treatment group was combined with thunder fire moxibustion on the basis of simple ovulation monitoring in the control group. After 6 months, it was found that the TCM syndromes in the treatment group were significantly improved, and the pregnancy rate was statistically significant compared with the control group (*P* < 0.05). Pan et al. [149] applied estradiol valerate combined with thunder fire moxibustion to treat patients with thin endometrium, and conducted a randomized controlled trial (RCT) on 100 patients with thin endometrial infertility. The control group was treated with estradiol valerate, while the experimental group was treated with thunder fire moxibustion on the basis of the control group. The changes in endometrial thickness, as well as the natural pregnancy rate and pregnancy time, were compared between the two groups before treatment and after 1, 2, and 3 months of treatment. And the results showed that after 1, 2, and 3 months of treatment, the endometrial thickness of the experimental group was thicker than that before treatment, and was significantly thicker than that of the control group. The natural pregnancy rate of the experimental group was significantly higher than that of the control group, and the pregnancy time (64.39 ± 11.77) *d* of the experimental group was significantly shorter than that of the control group (96.59 ± 15.34) *d*, suggesting that estradiol valerate combined with thunder fire moxibustion could promote endometrial growth, shorten the pregnancy time, and improve the rate of natural pregnancy.

*(3) Wheat Grain Moxibustion.* It is to knead moxa velvet into medium wheat grains with two pointed ends, which is glued to the acupoints and lit. When the patient feels unbearable heat, the moxa fire is quickly removed. It has the characteristics of supplementation and purgation, and biphasic regulation [150]. Li et al. [151] divided 80 patients with adenomyosis into two groups randomly. The control group was given levonorgestrel intrauterine contraceptive system treatment, and the observation group was given routine acupuncture and moxibustion with wheat grain. The treatment was performed once a day from 1 week before menstruation until menstrual cramps. The results showed that the uterine volume, endometrial thickness, inflammatory factors, and menstruation of adenomyosis patients were significantly improved after 3 months of treatment with wheat grain moxibustion combined with acupuncture. Xiao et al. [150] conducted a retrospective study on 60 patients with RIF treated with wheat grain moxibustion through data collection and telephone follow-up, and found that the application of wheat grain moxibustion to adjuvant treatment of RIF patients can increase the endometrial thickness on the endometrial transformation day in the hormone replacement cycle, reducing the endometrial preparation time before transplantation and improving the endometrial receptivity, thus increasing the pregnancy rate.

*(4) Sandwiched Moxibustion.*It is to separate moxa columns and skin with medicinal cakes, such as aconite, salt, ginger, and garlic, which have dual effects and mild stimulation on acupoints to produce biological effects of moxibustion and affect tissue and cell metabolism [152]. In the treatment of thin endometrial secondary infertility patients with sandwiched moxibustion combined with acupoint thread embedding, Gao et al. [153] believed that sandwiched moxibustion combined with acupoint thread embedding could not only exert the therapeutic effect of Shenque (RN8), but also prolong the stimulation time of acupoint thread embedding and enhance the therapeutic effect. In addition, Lin et al. [154] found that the use of EA plus ginger-partitioned moxibustion combined with Western medicine could also improve female endometrial receptivity, increase clinical pregnancy rate, and reduce early miscarriage rate.

*(5) Other Moxibustion.* Yang et al. [155] adopted the method of regulating Chong, boosting qi and invigorating the kidney combined with the Ren Mai moxibustion treatment in 80 patients with thin endometrial infertility, moving slowly between the Shenque (RN8) and Qugu (RN2) acupoints in the middle of the abdomen of the Ren Mai, which has the functions of regulating qi and blood, warming and nourishing the uterus, and promoting the blood circulation of the uterus. When the uterus is full of qi and blood, and the yin and yang are in harmony, pregnancy is achieved through the adjustment of meridians. Tao et al. [156] found that heat-sensitive moxibustion combined with acupoint injection could increase endometrial thickness, reduce uterine artery blood flow resistance, and improve endometrial receptivity. We have listed some RCTs in Table 5.

#### 2.2.12. Therapeutic Mechanisms of Moxibustion Therapy

Hu et al. [134] found that wheat grain moxibustion and estrogen treatment on “Guanyuan (RN4)” and “Shenshu (BL23)” acupoints could improve the expression levels of keratin, vimentin, VEGF, HOXA-10, LIF, and other related factors, induce angiogenesis, and improve endometrial receptivity by promoting the growth of endometrial epithelial cells and stromal cells; then, the endometrium gets better repaired. Pinopodes are considered to be the morphological markers of endometrial receptivity, whose region and tip are consistent with the implantation position of animal embryos, while the expression level of pinopodes in RIF patients is almost zero [157]. The mechanism of warm acupuncture may be related to its upregulation of endometrial tissue-related proteins and their mRNA levels. By increasing the expression of pinopodes, it can improve hemorheology in RIF patients, thereby improving the poor state of endometrial receptivity and promoting embryo implantation and increasing clinical pregnancy rate [143].

At present, the mechanisms of moxibustion have not been thoroughly studied in domestic and abroad, so more clinical and basic experiments are needed to verify and clarify its exact mechanisms (see Table 4).

### 2.3. Other Therapies

In addition to the above-mentioned oral CHM and acupuncture therapy, which can be used to treat infertility caused by endometrial factors, there are also some promising therapies, such as TCM retention enema, NMES, and photobiomodulation therapy, which are worthy of being further studied.

#### 2.3.1. Retention Enema of Traditional Chinese Medicine

After suffering from long-term infertility and repeated IVF-ET failures, TCM retention enema therapy has been tried by more and more women due to its unique advantages of trauma, minimal side effects, and simple operation. The special physiological structure of the female rectum adjacent to the uterus can be preserved by enema to promote the drug into the blood circulation and then absorbed through the rectal mucosa, permeating into the pelvic cavity and helping to reduce inflammation [158]. As the basis for implantation of zygote, the thickness and receptivity of endometrium are the key factors for successful implantation of embryos [159].

Pan et al. [160] conducted an RCT of TCM retention enema combined with EA on 60 patients with thin endometrial infertility, and the curative effect is obvious. The result indicated that the combination of the two methods could improve the local blood flow index of the endometrium, increase the endometrial thickness and receptivity, and then improve the pregnancy rate. As a classical prescription for treating infertility, channel-warming decoction can effectively improve the endometrial morphology of patients and improve the success rate of pregnancy [161], which was validated in a clinical trial of infertile patients with inadequate endometrial receptivity. The control group was given oral estradiol valerate alone, while the treatment group was given channel-warming decoction retention enema on the basis of the control group. After two menstrual cycles, the pregnancy rate in the treatment group reached 63.3%, while that in the control group was 33.3%; the difference was statistically significant (*P* < 0.05) [162]. Clinical enema treatment for infertile patients caused by adenomyosis is based on the theory that rectal administration of drugs can reduce inflammatory infiltration of pelvic tissue and improve the pelvic microenvironment [163]. In addition, TCM retention enema is also effective in treating infertility caused by endometritis. Jia et al. [164] conducted a clinical trial of antibiotics combined with TCM retention enema in patients with IVF failure and endometritis, and found that compared with the control group without any treatment and the experimental group treated only with antibiotics, its clinical pregnancy rate and embryo implantation rate were significantly increased.

### 2.4. Neuromuscular Electrical Stimulation

NMES is a low-frequency electrical therapy that targets nerve fibers with electrical pulses of different frequencies to activate an electrical potential, and induces nerve or muscle contraction [165, 166]. At present, it is mainly used as one of the means to treat infertility and pelvic floor muscle rehabilitation in female patients, and its application in infertility is mainly aimed at patients with thin endometrium. Zhu [167] explored the efficacy of NMES combined with Kuntai capsule for thin endometrial infertility patients who were treated with long-term estrogen therapy and had poor results. The results showed that the combination therapy could not only effectively increase endometrial thickness, but also regulate sex hormone levels and uterine hemodynamics, thereby improving clinical symptoms. In the treatment of thin endometrial infertility patients, He et al. [168] combined low-intensity focused ultrasound acupoint stimulation on the basis of biomimetic electrical stimulation for treatment. The studies have shown that it could improve endometrial blood perfusion, thus improving the shape and receptivity of the endometrium and increasing the clinical pregnancy rate.

The existing clinical trial results show that NMES is a kind of physiotherapy, which can obtain better curative effect, but its long-term curative effect needs to be further studied.

### 2.5. Photobiomodulation Therapy

Laser therapy is a photobiomodulation therapy that improves microcirculation by stimulating its own repair mechanisms to promote tissue healing, regeneration, and recovery. After laser treatment of cells, the energy generated is absorbed by it and increases the levels of ATP in cells, triggering and accelerating the rate of cell proliferation and differentiation [169, 170].

Studies have shown that about two thirds of infertile patients with repeated embryo implantation are due to endometrial dysreceptivity [171]. In a prospective randomized trial, Tsai et al. [172] pretreated 29 women with He–Ne laser irradiation before frozen-thawed embryo transfer, and the remaining 31 women did not receive any pretreatment. The results showed that He–Ne laser irradiation could improve endometrial microcirculation and increase endometrial receptivity and pregnancy rate by promoting the release and expression of growth factors and cytokines in the endometrium during implantation. Low-level laser therapy (LLLT) is also a photobiometric therapy that can absorb lasers at the electronic level without producing thermal effects [173]. El Faham et al. [174] conducted in vitro culture of 40 infertile women's endometrium to explore whether LLLT could enhance its proliferative ability. The studies have shown that when the wavelength of LLLT is 635 nm, the expression of endometrial receptivity genes can be induced, and the ability of endometrial cells to differentiate and regenerate is the strongest.

At present, photobiomodulation therapy is still an emerging treatment method in the stage of exploration, which has not been incorporated into mainstream medicine. In addition, the optimal wavelength and duration of laser irradiation are individual, so more comprehensive and systematic studies are needed to clarify the efficacy.

### 2.6. Improvement of Reproductive Tract Microbiota Disorders

With the rapid development of sequencing technology, more and more studies have been devoted to exploring the microbiota of the female reproductive tract. The female reproductive tract microbiota is mainly composed of bacteria, viruses, and other microorganisms, which distribute in the reproductive tract and participate in the immune and barrier processes of the body. Sequencing of microbial 16S rRNA genes has confirmed that the microbiota colonizes the entire female reproductive tract, which is not limited to the lower reproductive tract [175, 176]. Dysregulation of the microflora can trigger mechanisms such as inflammation and immune responses, which can lead to infertility by affecting embryo implantation [177]. Infertility caused by chronic endometritis is mainly due to the long-term inflammatory state of endometrium caused by the disorders of uterine microflora, which reduces endometrial receptivity and interferes with blastocyst development [178]. Compared with healthy women, infertile patients with chronic endometritis have lower vaginal microbial diversity and abundance, especially Lactobacillus, which determines the embryo implantation rate and pregnancy rate [179–181]. Therefore, many studies have begun to focus on increasing the abundance of Lactobacillus to improve pregnancy outcome in infertile patients, and probiotics have received more and more attention for their anti-inflammatory, immunomodulatory, and maintenance of healthy and safe reproductive system properties [182]. A study conducted by Kyono et al. [183] showed that the simultaneous use of antibiotics and probiotic supplements could effectively establish Lactobacillus predominance in the endometrium of infertile women to a certain extent, which is of great significance for improving the microbial status of the endometrium. Kadogami et al. [184] found that the probiotic vaginal suppository combined with the antibiotics group had the highest clinical response rate after grouping 329 patients. This prospective study demonstrated that probiotics combined with antibiotic therapy were effective and the Lactobacillus predominance could positively affect pregnancy outcome.

Currently, there are limited data on the mechanism and clinical application of probiotics to improve reproductive tract microbiota disorders, and the problems of treatment standards are still inconsistent. However, with the in-depth research on the microbiota, it is reasonable to believe that probiotics can provide new ideas and methods for the treatment of infertility.

#### 2.6.1. Vitamin D

Vitamin D is a fat-soluble vitamin synthesized by the skin after being irradiated by the solar ultraviolet, including two forms vitamin D_2_ and D_3_. 25-Hydroxyvitamin D (25(OH)D) is the final product of its cycle, which is also the active form of vitamin D. Vitamin D can not only play an important role in protecting bone and maintaining calcium/phosphorus homeostasis, but has also been found to be expressed in uterus and ovary, as well in more and more studies, indicating that vitamin D is also involved in regulating female reproductive activities [185–189].

In a cross-sectional study, the values of 25(OH)D and endometrial thickness were significantly increased in the pregnancy group after intracytoplasmic sperm injection, suggesting that 25(OH)D deficiency in women could affect endometrial thickness [190]. Ashour et al. [191] supplemented vitamin D to a rat model of vitamin D deficiency and found that vitamin D improved the endometrial receptivity of rats by adjusting the expression of HOXA-10. Additionally, a systematic review has investigated the relationship between vitamin D and the success rate of embryo transfer, and found that women with more adequate vitamin D showed higher clinical pregnancy rate and that higher vitamin D levels could improve the success rate of ART [192]. This will bring more hope to infertile women.

### 2.7. Dietary Intervention

Undoubtedly, as research progresses deeply, it is found that healthy eating patterns are closely related to female fertility. The Mediterranean diet is favored by women suffering from infertility due to its large intake of dietary fiber, fatty acids, and plant-based proteins [193]. Studies have shown that fatty acids are one of the important substrates in the reproductive process, and linoleic acid belongs to polyunsaturated fatty acids (PUFAs), which mainly affects fertility by improving the receptivity of endometrium and participating in embryo implantation [194–196]. A prospective study showed that North American women with a low intake of omega-3 PUFAs had lower fertility [197]. An RCT found that higher intakes of PUFAs, especially linoleic acid and omega-6 PUFAs, were associated with higher pregnancy rate in infertile women undergoing in vitro fertilization [198]. Appropriate supplementation of B vitamins can also improve endometrial receptivity and affect pregnancy outcome [199]. In addition, phytoestrogens may also have some impacts on female fertility. Soy isoflavones are nonsteroidal compounds present in soy that are similar to endogenous estrogens, which can increase endometrial thickness when given in appropriate doses [200, 201]. Similarly, increasing the intake of whole grains may also increase endometrial thickness, which can help improve pregnancy success [202, 203].

Caffeine intake is also seen as a potential factor affecting female reproductive performance [194]. Qian et al. [204] treated preimplantation mice with caffeine or transplanted normal blastocysts into the uterus of caffeine-treated nonpregnant mice, and both obtained abnormal embryo implantation results. It was suggested that caffeine may lead to impaired endometrial receptivity and pregnancy loss by interfering with the response of the uterine epithelium to steroid hormones. Although the effect and mechanism of caffeine on endometrial receptivity are still unclear at this stage, this study may provide some reference value for it.

### 2.8. Health Education Intervention

Infertile women often face dual pressure from family and society, and are more prone to tension, anxiety, and even depression. Therefore, it is of great significance to relieve the psychological pressure of patients so as to play an auxiliary role in the treatment of this disease. Health education is mainly to improve patients' cognition of disease and self-behavior management ability and adjust emotions reasonably through social support, empathy, health education, and other ways [205, 206]. In an RCT conducted by Luo et al. [207], 228 infertile women were divided into two groups: the observation group received health education intervention on the basis of clinical treatment, and the control group received general nursing intervention. After 6 months of treatment, it was found that the endometrial thickness of two groups increased, and the curative effect of the observation group was significantly better than that of the control group. The results showed that health education intervention based on clinical treatment could thicken the endometrium better and improve its blood flow indicators. At the same time, it can also improve patients' cognition of the disease, and then improve treatment compliance. Additionally, studies have shown that health education for IVF-ET infertile women and their families can help patients reduce negative emotions, improve their quality of life, and have a positive impact on pregnancy outcome [208]. More and more clinicians realize that health education intervention can help patients establish a good psychological state and improve the pregnancy rate.

In addition, acupoint sticking can stimulate the acupoints and meridians, as well as regulate the qi and blood of Chong and Ren. Combined with the warming effect, it promotes local blood circulation and plays a role in the treatment of infertility caused by adenomyosis [163]. Studies have shown that the hypoxic microenvironment also affects female reproductive capacity, which can result in failing to provide adequate oxygen to implantation failure after embryo transfer in patients with adenomyosis. However, there are no research reports on the relationship between hypoxic microenvironment and endometrial receptivity in infertile patients with adenomyosis [209, 210].

## 3. Results

From the data collected so far, CAM has a unique advantage in the treatment of infertility caused by endometrial factors. No matter TCM, acupuncture, or other auxiliary treatment methods, such as TCM retention enema, NMES, photobiomodulation therapy, and dietary intervention, they can all improve endometrial receptivity, increase endometrial thickness, or improve the local or overall inflammatory state of endometrium, then play a better interventional and treatment effect on this disease.

## 4. Discussion

In conclusion, CAM therapy has certain advantages in the treatment of infertility caused by endometrial factors. Although it is not the main intervention and treatment measure for infertility caused by endometrial factors, it is still being more widely used because it can restore the physiological function of endometrium, improve the pregnancy rate, adjust the psychological state of women, and improve the quality of life. However, CAM also has some limitations and lots of challenges at present.

Firstly, although the effectiveness of CAM in the treatment of infertility caused by endometrial factors has been confirmed by many studies, most of the studies have small sample sizes, which may cause deviations in statistical analysis. Furthermore, high-quality evidence-based evidence is often difficult to obtain, that is why it has not been recognized and promoted by many guidelines.

Secondly, as the main body of CAM in the treatment of this disease, though TCM and acupuncture therapy have obvious advantages, there are some differences in treatment among physicians, such as the composition and dosage of drugs, the choice of acupuncture points, and the frequency and intensity of acupuncture. It lacks high effectiveness from an evidence-based medicine perspective as well. In addition, nondrug CAM therapy also has problems such as inconsistent dosage and lack of standardized guidance, which limit the promotion and application of CAM to a certain extent.

Therefore, in order to solve the problems above better, more high-quality scientific evidence-based studies are urgently needed to confirm the efficacy and safety of CAM in the treatment of infertility caused by endometrial factors. How to explore evidence-based medicine and provide high-quality clinical evidence for it, how to standardize and unify nondrug CAM therapy, and how to accurately and effectively utilize different treatment methods in CAM are still the bottlenecks and challenges faced by CAM in the treatment of infertility caused by endometrial factors at present and for a long time in the future.

## Figures and Tables

**Table 1:** Summary of a randomized clinical trial of CHM.

Prescription	Design	Sample size	Interventions	Main outcomes	References
Gushen Antai pill	RCT	83	Treatment group: estradiol valerate + Gushen Antai pill	1. Total efficiency: treatment group—84.85% (28/33); control group—68.00% (34/50)	[29]
			Control group: estradiol valerate	2. Endometrial thickness after treatment: treatment group—0.73 ± 0.16 mm; control group—0.88 ± 0.18 mm	

Bushen Huoxue recipe	RCT	60	Treatment group: estradiol valerate + Bushen Huoxue recipe	1. Pregnancy rate: treatment group—43.3% (13/30); control group—26.7 (8/30)	[30]
			Control group: estradiol valerate	2. Endometrial thickness after treatment: treatment group—9.6 ± 1.0 mm; control group—8.2 ± 0.4 mm	

Bushen Yanggong decoction	RCT	120	Treatment group: Femoston + Bushen Yanggong decoction	1. Pregnancy rate: treatment group—53.33% (32/60); control group—31.67% (19/60)	[31]
			Control group: Femoston	2. Endometrial thickness after treatment: treatment group—8.19 ± 0.83 mm; control group—7.17 ± 0.82 mm	

Bubao decoction	RCT	115	Treatment group: Bubao decoction + aspirin enteric-coated aspirin group	1. Clinical pregnancy rate: treatment group—47.62%; aspirin group—32.56%	[34]
			Control group	2. Endometrial thickness after treatment: treatment group—10.65 ±3.03 mm; aspirin group—8.87 ± 2.50 mm	

Danyu decoction	RCT	50	Treatment group: Danyu decoction	1. Pregnancy rate: treatment group—60%; control group—28%	[37]
			Control group: vitamin *E* capsule	2. The rate of type A endometrium: treatment group—84%; control group—68%	
				3. Treatment group: RI—0.55 ± 0.08; PI—1.83 ± 0.61; control group: RI—0.64 ± 0.16; PI—2.19 ± 0.54	

Jieyuyubao pills	RCT	107	Treatment group: Jieyuyubao pills + clomiphene citrate	1. Pregnancy rate: treatment group—24.07%; control group—11.32%	[38]
				2. The rate of type A endometrium: treatment group—53.7%; control group—35.8%	
			Control group: clomiphene citrate	3. Symptom scores: treatment group—2.1 ± 0.6; control group—5.1 ± 0.6	

Erzhi Tiangui granule	RCT	66	Treatment group: Erzhi Tiangui granule + gonadotropin therapy	1. Clinical pregnancy rate: treatment group—54.55% (18/33); control group: 30.30% (10/33)	[39]
			Control group: gonadotropin therapy + placebo granules	2. Endometrial DNMT1 expression: treatment group—3.31 ± 0.46; control group—2.97 ± 0.49	

Yulin Zhuyun prescription	RCT	150	Combine group: Yulin Zhuyun prescription + clomifene citrate capsules	1. Clinical pregnancy rate: combine group—59.1% (26/44); CHM group—44.2% (19/43); Western medicine group—26.8% (11/41)	[40]
			CHM group: Yulin Zhuyun prescription	2. Integrin *αvβ*3: combine group—13.1 ± 2.67; CHM group—13.82 ± 2.04; Western medicine group—7.05 ± 1.37	
			Western medicine group: Clomifene citrate capsules	3. GLUT4: combine group—146.82 ± 21.84; CHM group—113.64 ± 15.62; Western medicine group—112.92 ± 18.54	

Kuntai capsule	RCT	71	Treatment group: Kuntai capsule + clomiphene	1. Pregnancy rate: treatment group—25% (9/36); control group—11.4% (4/35)	[41]
			Control group: placebo + clomiphene	2. Integrin *β*3: treatment group—1.78 ± 0.226; control group—1.46 ± 0.252	

Wenjing Quyu prescription	RCT	72	Treatment group: Wenjing Quyu prescription + Gn + HCG	1. Pregnancy rate: treatment group—59.38% (19/32); control group—12.96% (7/24)	[45]
			Control group: Gn + HCG	2. Covered pinopode area: treatment group—>50%; control group—<20%	

Huayu Xiaozheng prescription	RCT	60	Treatment group: Huayu Xiaozheng prescription + urofollicle-stimulating hormone + chorionic gonadotropin + letrozole	1. Pregnancy rate: treatment group—60% (18/30); control group—33.33% (10/30)	[52]
			Control group: urofollicle-stimulating hormone + chorionic gonadotropin + letrozole	2. Uterine volume: treatment group—100.27 ± 2.13 cm^3^; control group—102.47 ± 3.90 cm^3^	
				3. Endometrial thickness after treatment: treatment group—9.89 ± 044 mm; control group—8.04 ± 1.28 mm	

Yishen Sanjie Quyu decoction	RCT	102	Treatment group: Yishen Sanjie Quyu decoction	1. Uterine volume: treatment group—95.46 ± 3.21 cm^3^; control group—105.44 ± 4.23 cm^3^	[54]
			Control group: Sanjie Zhentong capsule	2. Symptom scores: treatment group—5.34 ± 1.46; control group—11.21 ± 2.01	

Wenjing Yangxue decoction	RCT	70	Treatment group: Wenjing Yangxue decoction + dydrogesterone tablets + moxibustion	1. Uterine volume: treatment group—136.47 ± 23.71 cm^3^; control group—152.38 ± 39.67 cm^3^	[55]
			Control group: dydrogesterone tablets + moxibustion	2. RI : treatment group—0.63 ± 0.07; control group—0.66 ± 0.05	

Penning granules	RCT	58	Treatment group: Penning granules	1. Pregnancy rate: treatment group—54.55% (18/33); control group—20% (1/25)	[58]
			Control group: levofloxacin + metronidazole tablets	2. CD38 : treatment group—8.89 ± 7.45; control group—20.12 ± 12.35	
				3. HIF-1*α* : treatment group—1.44 ± 0.95; control group—2.55 ± 1.40	

**Table 2:** Summary of basic studies of CHM.

Prescription	Experimental type	Sample size	Interventions	Main outcomes	References
Dingkun pill	CD1 mice	57	Dingkun pill (DK) group Control group Ovariectomized model group Delayed implantation model group	1. Embryo implantation rate (at 22:00 on D4) : DK-treated group—69% (20/29); control group—28.6% (8/28)	[60]
				2. Relative mRNA level: estrogen-target epithelial genes (Lif, Ltf) and stromal genes (Igf1, Spon2) were more induced in the DK group compared with the control	

Ankun Zhongzi Wan	Kunming mice	60	Ankun Zhongzi Wan (AZW) group	1. Pregnancy rate: AZM group—75% (15/20); model group—35% (7/20)	[61]
			Model group	2. ER : AZM group—10.55 ± 5.23; model group—4.31 ± 2.39	
			Normal group	3. PR : AZM group—8.73 ± 1.16; model group—2.91 ± 0.78	

Bushen Yiqi Huoxue recipe	Wistar rats	60	Bushen Yiqi Huoxue recipe (BYHR) group	1. Pregnancy rate: BYHR group—66.67%; model group—45.45%	[62]
			Progesterone group	2. P : BYHR group—118.98 ± 10.77; model group—73.62 ± 10.24	
			Model group	3. PR : BYHR group—0.23 ± 0.025; model group—0.14 ± 0.022	

Tiaojing Zhuyun capsule	Kunming mice	48	Tiaojing Zhuyun capsule (TZC) group Model group	1. E2 : TZC group—4.67 ± 1.25; model group—1.33 ± 0.80	[63]
				2. P : TZC group—102.15 ± 45.74; model group—50.18 ± 31.57	
				3. ER : TZC group–11.62 ± 3.74; model group—5.26 ± 2.11	
				4. PR : TZC group—8.07 ± 1.22; model group—2.76 ± 0.84	

Zhuyun I recipe	SD rats	70	Zhuyun I recipe (ZIR) group (7.3 g/kg, 14.6 g/kg) Model group Kidney deficiency and blood stasis group Normal group	1. E2 : ZIR group—21.8 ± 2.73(14.6 g/kg); 19.48 ± 6.05(7.3 g/kg); model group—11.00 ± 3.26	[64]
				2. P : ZIR group—75.41 ± 19.42(14.6 g/kg); 61.80 ± 14.19(7.3 g/kg); model group—39.88 ± 2.83	

Bushen Tiaoxue granules	SD rats	113	Controlled ovarian hyperstimulation (COH) model group Bushen Tiaoxue granules (BTG) + COH group (0.82 g/kg, 1.64 g/kg, 3.27 g/kg) Kunling Wan (KW) + COH group (0.46 g/kg, 0.91 g/kg, 1.82 g/kg) Control group	1. Pregnancy rate: COH + BTG (0.82 g/kg)—80% (8/10); COH + BTG (1.64 g/kg)—70% (14/20); COH + BTG (3.27 g/kg)—80% (12/15); COH + KW (0.46 g/kg)—92.9% (13/14); COH + KW (0.91 g/kg)—80% (12/15); COH + KW (1.82 g/kg)—80% (8/15)	[66]
Kunling Wan				2. Markers of blood vessels: the fluorescence intensity and the number of VEGFA and CD31‐positive vessels decreased in the COH group, while BTG and KW induced vascularization noticeably compared with the COH group.	

Bushen Huoxue recipe	Kunming mice	146	Control group Bushen Huoxue recipe group Controlled ovarian hyperstimulation (COH) model group Bushen Huoxue recipe (BSHX) group (5.7 g/kg, 11.4 g/kg, 22.8 g/kg) Bushen recipe group (5.7 g/kg) Huoxue recipe group (5.7 g/kg)	1. Pregnancy rate: BSHXR group (5.7 g/kg)—53.33% (10/27); BSHXR group (11.4 g/kg)—57.86% (19/28); BSHXR group (22.8 g/kg)—53.85% (7/13)	[67]
				2. Markers of blood vessels: HIF1*α*, VEGFA, and COX2-PGE2 level in the model group was lower than that in the control group, while BSHXR and BSR treatment could improve these levels	
Xiaoyao powder	Kunming mice	78	Controlled group COH group Xiaoyao (XYP) powder + COH group	Pregnancy rate: XYP + COH groups—65% (13/20); COH group—40% (8/20); controlled group—85% (17/20)	[68]

Tiaojing Cuyun recipe	Kunming mice	120	Control group Embryo implantation dysfunction (EID) model group Progesterone (Prog) + EID group Tiaojing Cuyun recipe (TJCYR) + EID groups (12 g/kg, 24 g/kg, 48 g/kg)	1. The number of implantation sites: Control group—15; EID group—2; TJCYR + EID (12 g/kg)—2; TJCYR + EID (24 g/kg)—10; TJCYR + EID (48 g/kg)—12	[69]
				2. Pinopodes were well-developed; they were sparse; and they in this group improved significantly following treatment with TJCYR.	

Wenshen Yangxue decoction	Wistar rats	100	Control group Model group Wenshen Yangxue decoction (WSYXD) groups (1.3/100 g, 2.6/100 g, 5.2/100 g)	1. Implantation rate: control group—100%; model group—40%; WSYX D (1.3/100g)—40%; WSYXD (2.6/100 g)—50%; WSYSD (2.6/100 g)—70%	[70]
				2. Control and high groups: a large number of pinopodes but little short microvilli on the endometrial surface; middle group: pinopodes existed in only parts of endometrium; low groups: no pinopode, but numerous microvilli can be found	

Zhuyun recipe	Kunming mice	139	Control group Ovarian stimulation (OS) model group OS + Zhuyun recipe group (ZYR) Embryo implantation dysfunction (EID) model group EID + Zhuyun recipe group Zhuyun recipe group	1. Pregnancy rate: control group—83.33%; OS model group—6.67%; OS + ZYR group—54.55%; EID model group—18.75%; EID + ZYR group—65.22%	[71]
				2. OS and EID model group: a moderate number of pinopodes without microvilli; OS + ZYR and EID + ZYR group: abundant fully developed pinopodes	

Yiqixue Buganshen recipe	Kunming mice	180	Control group Model group Treatment group: Yiqixue Buganshen recipe (YQBSR) Model group: 9.10 ± 0.93; Treatment group: 12.60 ± 0.73	1. Blastocyst implantation: control group—13.70 ± 0.67;	[77]
				2. Integrin *ανβ*3 expression in the treatment group was higher than in the model group (*P* < 0.05)	

Tokishakuyakusan	Wistar rats	Not clear	Tokishakuyakusan (TSS) group (1%, 3%) Model group Normal group	1. The number of Implantation: 1% TSS group—11.4 ± 1.4; 3% TSS group—14.7 ± 0.8; model group—9.0 ± 2.1	[78]
				2. LIF mRNA levels: model group—0.42 ± 0.05; 3%TSS group—2.40 ± 0.93	

Zishen Yutai pills	SD rats	90	Normal group, control group Zishen Yutai pill (ZYP) group	1. Pregnancy rate: control group—40%; ZYP group—70%	[79]
				2. The mRNA and protein expression levels of LIF in the ZYP group were significantly higher than those in the control group	

Modified Shoutaiwai recipe	Kunming mice	70	Modified Shoutaiwai recipe (MSTW) group Aspirin group Control group	1. Expression of integrin *β*3: MSTW group—46.7%; aspirin group—23.3%; control group—0%	[80]
				2. Expression of LIF mRNA : MSTW group—0.9835 ± 0.0059; aspirin group—0.9793 ± 0.0061; control group—0.9670 ± 0.0103	

Qiu's Neiyi recipe	ICR mice	45	Model group, danazol group Qiu's Neiyi recipe group (5 g/kg, 10 g/kg, 20 g/kg)	1. Qiu suppressed the expression of IL-1*β*, IL-6, and TNF-*α*	[84]
				2. The expression of these proteins was significantly decreased after being treated with qiu and danazol (*P* < 0.05).	

Modified Danggui Shaoyao powder	SD rats	60	Blank group Model group Gynecological Qianjin capsule group Modified Danggui Shaoyao Powder (MDSP) group (6.48 g/kg , 12.96 g/kg, 25.92 g/kg)	1. NF-*κ*B : model group—0.72 ± 0.23; GQJC group—0.59 ± 0.20; MDSP group (6.48 g/kg)—0.60 ± 0.03; MDSP group (12.96 g/kg)—0.56 ± 0.10; MDSP group (6.48 g/kg)—0.46 ± 0.23	[85]
				2. TGF-*β*1: model group—2.54 ± 3.88; GQJC group—1.57 ± 1.78; MDSP group (6.48 g/kg)—1.54 ± 1.35; MDSP group (12.96 g/kg)—1.33 ± 1.32; MDSP group (6.48 g/kg)—1.10 ± 1.08	

Shaofu Zhuyu decoction	SD rats	46	Control group Model group Estradiol valerate group Shaofu Zhuyu decoction (SZD) group (144 mg/kg, 288 mg/kg)	Embryo implantation rate: model group—33.33%; estradiol valerate group—55.56%; SZD group (144 mg/kg)—44.44%; SZD group (288 mg/kg)—88.89%	[87]

Bushen Tiaochong decoction	SD rats	60	Normal control group; model group; estradiol valerate group; Bushen Tiaochong decoction group (8.525 g/kg, 17.05 g/kg, 34.10 g/kg)	Compared with the model group, the endometrium of each administration group,caspase-1, IL-1*β*, IL-18, GSDMD, and their mRNA expression significantly decreased low (from *P* < 0.05 to *P* < 0.01)	[88]

**Table 3:** Summary of a randomized clinical trial of acupuncture.

References	Design	Sample size	Interventions	Main outcomes	Acupuncture points
[101]	RCT	60	Treatment group: acupuncture + TCM Control group: estradiol valerate tablets	1. Total efficiency: treatment group—26.7% (8/30); control group—6.7% (2/30) 2. Endometrial thickness after treatment: treatment group—1.071 ± 0.144 mm; control group—1. ± 0.150 mm	Guanyuan (RN4), Sanyinjiao (SP6) Shenshu (BL23), Zigong (EX-CA1)

[102]	RCT	60	Treatment group: acupuncture + letrozole tablets Control group: letrozole tablets	1. Total efficiency: treatment group—66.7% (20/30); control group—40.0% (12/30) 2. Endometrial thickness after treatment: treatment group—10.32 ± 1.77 mm; control group—9.31 ± 1.47 mm	Baihui (DU20), Mingmen (DU4) Geshu (BL17), Ganshu (BL18) Shenshu (BL23), Ciliao (BL32) Guanyuan (RN4), Qihai (RN6) Dahe (KI12), Sanyinjiao (SP6) Gongsun (SP4), Daimai (GB26)

[103]	Single-blind RCT	60	Treatment group: acupuncture + estradiol valerate tablets Control group: estradiol valerate tablets	1. Total efficiency: treatment group—63.33% (19/30); control group—33.33% (10/30) 2. Endometrial thickness after treatment: treatment group—0.98 ± 0.33 mm; control group—0.68 ± 0.22 mm	Zhongwan (RN12), Tianshu (ST25) Daimai (GB26), Guanyuan (RN4) Qihai (RN6), Zhongji (RN3) Zigong (EX-CA1), Xuehai (SP10) Zusanli (ST36), Sanyinjiao (SP6) Taichong (LV3), Mingmen (DU4) Shenshu (BL23), Ganshu (BL18) Yaoyangguan (DU3),Yaoshu (DU2) Guanyuanshu (BL26)

[104]	RCT	90	Treatment group: acupuncture + TCM + aspirin enteric-coated tablets Control group: aspirin enteric-coated tablets	Endometrial thickness after treatment: treatment group—10.59 ± 2.25 mm; control group—5.39 ± 1.00 mm	Pishu (BL20), Shenshu (BL23) Ciliao (BL32), Sanyinjiao (SP6) Shuiquan (KI5)

[105]	RCT	126	Treatment group: acupuncture + moxibustion + TCM + estradiol valerate tablets + progesterone capsules Control group: estradiol valerate tablets + progesterone capsules	1. Total efficiency: treatment group—47.6% (30/63); control group—28.6% (18/63) 2. Endometrial thickness after treatment: treatment group—7.99 ± 1.46 mm; control group—6.21 ± 1.28 mm	Guanyuan (RN4), Taixi (KI3) Sanyinjiao (SP6), Shenshu (BL23) Mingmen (DU4), Taichong (LV3) Xingjian (LV2)

[106]	RCT	72	Treatment group: acupuncture + warm group acupuncture + EA + estradiol valerate tablets Control group: estradiol valerate tablets	1. Total efficiency: treatment group—50.0% (18/36); control group—33.3% (12/36) 2. Endometrial thickness after treatment: treatment group—9.94 ± 1.04 mm; control group—7.92 ± 1.0 mm	Gongsun (SP4) Neiguan (PC6)

[108]	RCT	74	Treatment group: Tongyuan acupuncture + estradiol valerate tablets Control group: estradiol valerate tablets	1. Total efficiency: treatment group—37.8% (14/37); control group—16.2% (6/37) 2. Endometrial thickness after treatment: treatment group—9.61 ± 0.76 mm; control group—7.72 ± 0.51 mm	Qiangjian (DU18), Naohu (DU17) Dazhui (DU14), Baihui (DU20) Xinshu (BL15), Geshu (BL17) Ganshu (BL18), Shenshu (BL23) Ciliao (BL32), Weizhong (BL40) Yongquan (KI1), Yintang (EX-HN3) Zhongwan (RN12), Tianshu (ST25) Guanyuan (RN4), Qihai (RN6) Luanchao (TF2), Zigong (EX-CA1) Xuehai (SP10), Zusanli (ST36) Sanyinjiao (SP6)

[109]	Double-blind RCT	120	Treatment group 1: acupuncture test Treatment group 2: acupuncture control Control group: no intervention	Total efficiency: treatment group 1—58.69% (27/46) Treatment group 2—38.29% (18/47) Control group—33.33% (9/27)	Treatment group1: Guanyuan (RN4), Zhongji (RN3) Zigong (EX-CA1), Sanyinjiao (SP6) Guilai (ST29), Xuehai (SP10) Treatment group2: Fengshi (GB31), Yinlingquan (SP9) Waiguan (SJ5), Sidu (SJ9)

[110]	RCT	64	Treatment group: EA intervention Control group: no intervention	1. Total efficiency: treatment group—50.00% (15/30); control group—41.94% (13/31) 2. Endometrial thickness after treatment: treatment group—9.03 ± 1.68 mm; control group—9.46 ± 1.67 mm	Baihui (DU20), Zhongwan (RN12) Guanyuan (RN4), Qihai (RN6) Zigong (EX-CA1) Fenglong (ST40) Xuehai (SP10), Sanyinjiao (SP6) Ganshu (BL18), Shenshu (BL23)

[111]	RCT	80	Treatment group: EA + clomiphene citrate tablets Control group: clomiphene citrate tablets	1. Total efficiency: treatment group—21.1% (8/38); control group—16.2% (6/37) 2. Endometrial thickness after treatment: treatment group—8.21 ± 1.08 mm; control group—6.54 ± 1.12 mm	Qihai (RN6), Guanyuan (RN4) Dahe (KI12), Zigong (EX-CA1) Zhongji (RN3), Diji (SP8) Sanyinjiao (SP6), Shenshu (BL23) Sanjiaoshu (SP6), Ciliao (BL32)

[112]	RCT	80	Treatment group: TCM + EA + aspirin enteric-coated tablets Control group: aspirin enteric-coated tablets	1. Total efficiency: treatment group—40.0% (16/40); control group—27.5% (11/40) 2. Endometrial thickness after treatment: treatment group—8.78 ± 1.67 mm; control group—7.15 ± 1.42 mm	Guanyuan (RN4), Zigong (EX-CA1) Zhongji (RN3), Sanyinjiao (SP6) Xuehai (SP10), Zusanli (ST36) Taixi (KI3), Zhaohai (KI6) Qihai (RN6), Yongquan (KI1)

**Table 4:** Summary of basic studies of the acupuncture and moxibustion.

References	Animal type	Sample size	Interventions	Main outcomes	Acupuncture points
[118]	Wistar rats	60	Normal group Embryo implantation dysfunction model group Acupuncture group	1. Implantation rate: normal group—95% (19/20); model group—45% (9/20); acupuncture group—75% (15/20) 2. PR-positive staining in the acupuncture group was significantly higher than that in the model group.	Zusanli (ST36), Sanyinjiao (SP6) Taichong (LV3)

[124]	SD rats	Not clear	PCOS model group Clomiphene group Clomiphene + acupuncture group Control group	1. Endometrial thickness: model group—30 ± 21 *μ*m; clomiphene group—20 ± 27 *μ*m; clomiphene and acupuncture group—59 ± 31 *μ*m; control group—85 ± 23 *μ*m 2. The expression of ER, PR, HOXA10, LIF mRNA, LIF, and integrin *αvβ*3 protein in endometrium of group *C* + *A* increased.	Guanyuan (RN4), Zhongji (RN3) Sanyinjiao (SP6), Zigong (EX-CA1)

[125]	Wistar rats	40	Normal group (N) Embryo implantation dysfunction model group (M) Acupuncture group (A) Control group (C)	1. Pregnancy rate: N group—100% (10/10); M group—40% (4/10); A group—70% (7/10); C group—40% (4/10) 2. The expression level of VEGF mRNA in the acupuncture group was significantly higher than that in the model group and the control group.	Zusanli (ST36), Sanyinjiao (SP6)

[128]	C57BL6 mice	50	Blank control group Superovulation model group Erbuzhuyu decoction (EBZYD) group Acupuncture group EBZYD + acupuncture group	1. The endometrial thickness significantly increased in the EBZYD, acupuncture, and EBZYD combined with the acupuncture group compared with the model group. 2. The expression levels of HOXA10 and VEGF significantly increased in the EBZYD + acupuncture group compared with the EBZYD and acupuncture group.	Guanyuan (RN4), Sanyinjiao (SP6) Shenshu (BL23)

[129]	SD rats	40	Control group Thin endometrium model group BMSC group BMSC + EA group	The amount of ER and PR in the BMSC group and the combined group was significantly higher than that in the model group.	Guanyuan (RN4), Zigong (EX-CA1) Sanyinjiao (SP6)

[130]	SD rats	70	Normal group (G) PCOS model group (M) Clomiphene citrate group (CC) Clomiphene citrate + PVG group (CC + PGV) Clomiphene + EA group (CC + *A*) Clomiphene + Bushen Huoxue recipe group (CC + *M*) clomiphene citrate + acupuncture + medicine group (CC + *M* + A)	1. Endometrial thickness: G—92 ± 25 *μ*m; M—32 ± 20 *μ*m; CC—22 ± 16 *μ*m; CC + PGV—33 ± 19 *μ*m; C + M—32 ± 20 *μ*m; CC + A—52 ± 23 *μ*m; CC + *M* + A—89 ± 27 *μ*m 2. Compared with the model group, the mRNA expression of PR and HOXA10 in each group was higher in CC + *A*, CC + *M*, CC + *M* + A, and CC + PVG.	Guanyuan (RN4) Sanyinjiao (SP6) Zigong (EX-CA1)

[131]	Adult Sprague Dawley rats	60	Control group Thin endometrium model group EA group	1. Pregnancy rate: control group—100% (20/20); thin endometrium model group—20% (4/20); EA group—100% (20/20) 2. A significantly thicker endometrial lining was identified in the EA group than in the model group. 3. The protein and mRNA expression of HBEGF, Itgav, and Itg*β*3 was significantly upregulated in the EA group relative to that in the model group	Sanyinjiao (SP6) Zigong (EX-CA1) Guanyuan (RN4)

[132]	SD rats	80	Normal treatment group Controlled ovarian hyperstimulation (COH) model treatment group (Model) Low-frequency EA group (LF-EA) High-frequency EA treatment group (HF-EA)	1. The results showed that the thickness of endometrium in the LF-EA group and the HF-EA group was significantly higher than that in the model group. 2. The expressions of LIF and P-STAT3 in the LF-EA or HF-EA group were evidently higher than those in the model treatment group.	Guanyuan (RN4) Zusanli (ST36)

[133]	Kunming mice	60	Natural cycle group (NC) COH group EA group	After EA treatment, the expression of IGF-1 protein and its mRNA protein in mouse endometrium increased.	Guanyuan (RN4) Zhongji (RN3) Sanyinjiao (SP6)

[134]	SD rats	40	Normal group Endometrial model group Estrogen group Wheat grain moxibustion group	1. Endometrial thickness: normal group > wheat grain moxibustion group > estrogen group > endometrial model group 2. Grain moxibustion can improve endometrial receptivity by upregulating the expression of keratin, vimentin, and VEGF in rats' endometrium, and improving the levels of endometrial receptivity-related factors such as HOXA10 and LIF.	Shenshu (BL23) Guanyuan (RN4)

**Table 5:** Summary of a randomized clinical trial of moxibustion.

References	Design	Sample size	Interventions	Main outcomes	Acupuncture points
[144]	RCT	92	Treatment group: warm acupuncture Control group: estradiol valerate tablet.	1. Pregnancy rate: treatment group—34.78% (16/50); control group—13.04% (6/50) 2. Endometrial thickness after treatment: treatment group—9.89 ± 2.06 mm; control group—8.02 ± 2.03 mm	Guanyuan (RN4), Zigong (EX-CA1) Zhongji (RN3), Yinjiao (RN7)

[145]	RCT	136	Treatment group: warm acupuncture + Zhen qi decoction Control group: estradiol valerate.	1. Pregnancy rate: treatment group—63.24% (43/68); control group—41.18% (28/68) 2. Endometrial thickness after treatment: treatment group—9.37 ± 1.53 mm; control group—7.49 ± 1.38 mm	Guanyuan (RN4), Zhongji (RN3) Sanyinjiao (SP6), Zusanli (ST36)

[146]	RCT	56	Treatment group: warm acupuncture Control group: antibiotics and flexor progesterone.	1. Clinical pregnancy rate: treatment group—46.3%; control group—20.7% 2. Endometrial thickness after treatment: treatment group—9 ± 2 mm; control group—9 ± 3 mm	Zhongwan (RN12), Tianshu (ST25) Guanyuan (RN4), Zhongji (RN3) Zigong (EX-CA1), Liangqiu (ST34) Zusanli (ST36), Shangjuxu (ST37) Xiajuxu (ST39)

[148]	RCT	60	Treatment group: thunder fire moxibustion Control group: ovulation monitoring	Pregnancy rate: treatment group—50.00% (15/30); control group—23.30% (7/30)	Guanyuan (RN4), Qihai (RN6)

[149]	RCT	100	Treatment group: estradiol valerate and thunder fire moxibustion Control group: estradiol valerate	1. Pregnancy rate: treatment group—40.00% (20/50); control group—20.00% (10/50) 2. Endometrial thickness after treatment: treatment group—10.56 ± 2.88 mm; control group—7.86 ± 2.16 mm	Shenque (RN8), Guanyuan (RN4) Zigong (EX-CA1), Zhongji (RN3) Guilai (ST29), Qihai (RN6)

[150]	Retrospective study	60	Treatment group: wheat moxibustion Control group: hormone replacement cycle intima; corpus luteum.	1. Pregnancy rate: treatment group—33.33% (10/30); control group—23.33% (7/30) 2. Endometrial thickness after treatment: treatment group—9.28 ± 1.15 mm; control group—8.35 ± 1.14 mm	Qihai (RN6), Guanyuan (RN4) Zigong (EX-CA1), Zusanli (ST36)

[151]	RCT	80	Treatment group: conventional acupuncture and wheat grain moxibustion Control group: levonorgestrel	1. Total efficiency: treatment group—97.5% (39/40); control group—82.5% (33/40) 2. Endometrial thickness after treatment: treatment group—6.7 ± 0.7 mm; control group—7.6 ± 0.8 mm	Guanyuan (RN4), Zigong (EX-CA1) Zhongji (RN3), Sanyinjiao (SP6) Diji (SP8), Shiqizhui (EX-B8) Ciliao (BL32)

[154]	RCT	70	Treatment group: EA + ginger moxibustion isolation Control group: letrozole + HCG	1. Pregnancy rate: treatment group—56.25% (18/32); control group—30.3% (10/32) 2. Endometrial thickness after treatment: treatment group—0.89 ± 0.14 mm; control group—0.78 ± 0.10 mm	Zigong (EX-CA1), Guilai (ST29) Sanyinjiao (SP6), Taichong (LV3) Hegu (LI4), Qihai (RN6) Dahe (KI12), Luanchao (TF2) Zusanli (ST36), Taixi (KI3) Guanyuan (RN4), Shenque (RN8) Wushu (GB27), Zhongji (RN3)

[155]	RCT	80	Treatment group: invigorating qi-tonifying kidney Chinese medicine + Ren Mai moxibustion Control group: estradiol + dydrogesterone	1. Pregnancy rate: treatment group—67.5% (27/40); control group—42.5% (17/40) 2. Total efficiency: treatment group—87.5% (35/40); control group—65.0% (26/40)	Shenque (RN8), Qugu (RN2) Zhongji (RN3), Guanyuan (RN4)

[156]	RCT	210	Treatment group 1: heat-sensitive moxibustion + acupoint injection Treatment group 2: heat-sensitive moxibustion Control group: cefoxitin sodium	Endometrial thickness after treatment: treatment group 1—10.0 ± 0.98 mm; treatment group 2—9.24 ± 0.87 mm; control group—7.89 ± 1.02 mm	Yaoyangguan (DU3) Guanyuan (RN4), Qihai (RN6) Shenshu (BL23), Sanyinjiao (SP6) Yinlingquan (SP9), Zigong (EX-CA1)
